# Effects of benidipine, paracetamol, and their combination on postoperative and normal tissue pain thresholds

**DOI:** 10.3389/fphar.2023.1326128

**Published:** 2024-01-05

**Authors:** Zehra Bedir, Kezban Tuna Ozkaloglu Erdem, Omer Doymus, Halis Suleyman, Bulent Yavuzer, Betul Cicek, Durdu Altuner, Renad Mammadov, Mehmet Yilmaz, Taha Abdulkadir Coban, Bahadir Suleyman, Seval Bulut

**Affiliations:** ^1^ Department of Anaesthesiology and Reanimation, University of Health Sciences, Erzurum State Hospital, Erzurum, Türkiye; ^2^ Department of Anaesthesiology and Reanimation, University of Health Sciences, Antalya Training and Research Hospital, Antalya, Türkiye; ^3^ Department of Pharmacology, Faculty of Medicine, Erzincan Binali Yildirim University, Erzincan, Türkiye; ^4^ Department of Physiology, Faculty of Medicine, Erzincan Binali Yildirim University, Türkiye; ^5^ Department of Orthopaedics and Traumatology, Private Deva Hospital, Gaziantep, Türkiye; ^6^ Department of Biochemistry, Faculty of Medicine, Erzincan Binali Yildirim University, Erzincan, Türkiye

**Keywords:** benidipine, paracetamol, pain, rat, analgesic effect, proinflammatory cytokines

## Abstract

**Introduction:** In clinical practice, inadequate pain inhibition leads to increased morbidity and mortality. Increased intracellular calcium, oxidants, and proinflammatory cytokines are known to play a role in the pathogenesis of postoperative pain. Therefore, we investigated the analgesic effects of benidipine, paracetamol, and benidipine-paracetamol combination (BPC) on postoperative and normal pain thresholds in rats.

**Material and methods:** Sixty-four male albino Wistar rats weighing 285–295 g were used. The without-incision rats were divided into 4 subgroups: healthy control, benidipine alone, paracetamol alone, and BPC. The scalpel-incision rats were divided into 4 subgroups: scalpel incision, scalpel incision + benidipine, scalpel incision + paracetamol, and scalpel incision + BPC. Paw pain thresholds of rats were measured using a Basile algesimeter. Biochemical analyses were performed on the paw tissues of 6 rats randomly taken from the experimental groups, each containing 8 rats. Rats were sacrificed immediately after the measurements. After the pain threshold tests were finished, the paw tissues were removed and malondialdehyde (MDA), total glutathione (tGSH), cyclooxygenase (COX), and interleukin-6 (IL-6) levels were measured.

**Results:** There was no significant difference between the groups in paw pain threshold and measured biochemical parameters in rats without incision. The decrease in the pain threshold of the incised paw was also best prevented by BPC, followed by benidipine and then paracetamol. Furthermore, increases in scalpel-incised paw tissue MDA, COX-2, and IL-6 levels and the decrease in tGSH were significantly suppressed by benidipine and BPC, while paracetamol could only significantly inhibit the increase in IL-6 production.

**Conclusion:** The combination of the L-type Ca^2+^ channel blocker benidipine and paracetamol (BPC) may provide potent analgesia. Our experimental results support that BPC may be useful in the treatment of severe pain that cannot be adequately inhibited by paracetamol.

## 1 Introduction

The definition of pain in Merskey’s study in 1964 was the first to receive wide acceptance and was subsequently revised by the International Association for the Study of Pain in 1974 (Merskey, 1964). According to this definition, pain is a sensory and emotional experience associated with tissue damage or described in the context of that damage (Williams and Craig, 2016). Postoperative pain emerges as a normal response to a surgical intervention (Khan et al., 2011). Approximately 75% of patients complain of moderate to severe postoperative pain. Currently, opioid drugs are frequently used in the treatment of postoperative pain (Angster and Hainsch-Muller, 2005). However, opioid-related side effects such as nausea, vomiting, constipation, excessive sedation, somnolence and respiratory depression lead to dose limitation and inadequate analgesia (Rawal, 2016). Numerous nonopioid drugs, including paracetamol, NSAIDs, local anaesthetics, gabapentinoids, ketamine and glucocorticoids, are also used in postoperative pain management (Rawal, 2016). The simultaneous use of multiple analgesic drugs to target different analgesia mechanisms is aimed at reducing opioid-induced side effects and enhancing the analgesic effect through synergistic effects (Rawal, 2016). Up to 20 per cent of patients also require interventional pain management after surgery (Angster and Hainsch-Muller, 2005). Despite available treatments, evidence suggests that more than half of patients undergoing surgical procedures suffer from poorly controlled postoperative pain (Gorsky et al., 2021). In addition, many issues such as gastrointestinal side effects related to NSAIDs, cardiovascular problems related to COX-2 inhibitors, and concerns about abuse of gabapentinoids limit the use of current therapies (Angster and Hainsch-Muller, 2005; Rawal, 2016). In addition, non-postoperative pain in daily life occur in the form of head-neck, musculoskeletal, neuropathic, and cancer-related chronic pain (Treede et al., 2019). In clinical terms, insufficiently inhibited pains can lead to pulmonary, cardiac, and renal function disorders (Soto and Fu, 2003; Ochroch and Gottschalk, 2005). This in turn results in decreased patient satisfaction and increased morbidity and mortality (Lovich-Sapolo et al., 2015). Increased production of malondialdehyde (MDA) and a decrease in reduced glutathione with oxidative stress are implicated in postoperative pain (Onk et al., 2018). In addition, postoperative pain has also been linked to such proinflammatory cytokines as interleukin 1 beta (IL-1β) and tumour necrosis factor alpha (TNF-α), which directly stimulate primary sensory neurons (Ji et al., 2014; Song et al., 2016). Cetin et al. also showed the role of cyclooxygenase-2 (COX-2) in the pathogenesis of postoperative pain (Cetin et al., 2016). COX-2 is an enzyme responsible for inflammation (Suleyman et al., 2007). The increase in COX-2 activity is directly proportional to the increase in intracellular calcium (Suleyman and Ozcicek, 2019). Calcium channels constitute another mechanism underlying postoperative pain. Research has confirmed that low-voltage-activated calcium channels increase the excitability of sensory neurons following surgical incisions in rats (Joksimovic et al., 2018). The literature shows that calcium channel activation, an increase in oxidants and proinflammatory cytokines, and a decrease in antioxidants are associated with both postoperative and non-postoperative pain (Kuyrukluyildiz et al., 2016; Joksimovic et al., 2019).

Benidipine, the effect of which against postoperative and normal tissue pain thresholds is investigated in this study, is a second-generation dihydropyridine derivative and antihypertensive drug that blocks L-, N-, and T-type calcium channels (Kosaka et al., 2010; Yang et al., 2019; Kocak et al., 2021). In addition, benidipine is known to reduce myocardial infarction and post-ischaemia/reperfusion (I/R) oxidative stress in mice (Ohtani et al., 2012). Cakır et al. showed that benidipine protects brain tissue against I/R damage by inhibiting the overproduction of COX-2 (Cakir et al., 2021). Benidipine has also been reported to produce an anti-inflammatory effect by stabilizing the production of proinflammatory cytokines such as IL-1β and TNF-α (Unlubilgin et al., 2017).

Paracetamol (acetaminophen, N-acetyl-p-aminophenol) is another drug whose effect on postoperative and normal tissue pain thresholds is investigated in this study. Paracetamol is an analgesic and antipyretic agent widely used around the world (Shama and Mehta, 2014). It is used alone or in combination with other analgesics to treat pain associated with acute and chronic conditions and generally to reduce opioid requirements (Authors Anonymous, 2018). The effect mechanism of paracetamol has not been satisfactorily explained; however, paracetamol has been shown to exhibit an inhibitory effect on COX-1 and COX-2 activity in peripheral tissues, although not to the same extent (Jozwiak-Bebenista and Nowak, 2014).

Information from the literature suggests that benidipine and paracetamol can be effective in the treatment of postoperative pain. However, our scan of the literature revealed no studies investigating the effects of benidipine, paracetamol, and a combination thereof (BPC) on postoperative and normal tissue pain thresholds. The purpose of this study was therefore to investigate the effects of benidipine, paracetamol, and BPC on experimentally induced postoperative and normal pain thresholds in rats.

## 2 Materials and methods

### 2.1 Animals

Sixty-four male albino Wistar rats weighing 285–295 g were used in this study. All rats were obtained from the Erzincan Binali Yıldırım University Experimental Animals Application and Research Center, Türkiye. Prior to the experiment, the rats were housed under appropriate laboratory conditions (22°C) in a 12/12 h light/dark cycle. The protocols and procedures were approved by Erzincan Binali Yildirim University Animal Experimentation Ethics Committee (Meeting Date: 29.11.2022; Meeting Number: 2022/11; Decision Number: 58).

### 2.2 Chemicals

Benidipine was obtained from Deva Drug Co. (Türkiye), paracetamol from Sanofi Aventis (Türkiye), and sodium thiopental from, I.E., Ulagay (Türkiye).

### 2.3 Experimental groups

#### 2.3.1 Without-incision tissue pain test groups

The animals in this group were assigned into 4 subgroups as healthy control (HC), benidipine application alone (BN), paracetamol application alone (PC), and BPC (BPCG).

#### 2.3.2 Postoperative pain test groups

The animals in the postoperative pain test were divided into 4 groups–a control group subjected to a scalpel incision to the paw only (SIC) (Williams and Craig, 2016), a scalpel incision + benidipine (SIB) group, a scalpel incision + paracetamol (SIP) group, and a scalpel incision + BPC (SIBPC) group.

### 2.4 Experimental procedure

#### 2.4.1 Effects of benidipine, paracetamol, and BPC on postoperative pain threshold test

The postoperative pain model in rats was applied using a known and widely employed method (Kara et al., 2010). Briefly, a scalpel was used to make a transverse subcutaneous incision to the left hind paw in all rats. The margins of the incisions were then sutured with 5–0 silk. Paracetamol and benidipine tablets were first pulverized with a mortar and pestle and then suspended in distilled water as solvent. Twenty-4 hours after the operation, the SIB group (n = 8) received 4 mg/kg benidipine by oral gavage, the SIP group (n = 8) 500 mg/kg paracetamol, and the animals in the BPCG group (n = 8) 4 mg/kg benidipine plus 500 mg/kg paracetamol. Distilled water was administered as a solvent to the SIC group (n = 8) the same way. At the third hour after drug administration, the paw pain thresholds of all rats in all groups were measured using a Basile algesimeter (Ugo Basile, Italy) (Cadirci et al., 2010)**.** For the determination of paw pain thresholds, rats were placed on the device and waited for 10–15 min to calm down. Then, the stimulus probe was placed on the plantar surface of the left hind paw and the device was switched on. Increasing force was applied to the plantar surface. The device recorded the value at the moment the animal pulled its hind paw. The arithmetic mean of three consecutive measurements was taken to determine this value. After the measurements of all groups were completed, the rats were sacrificed with high-dose sodium thiopental (50 mg/kg), and the left hind paw tissues were removed. Oxidant, antioxidant, proinflammatory cytokine, and COX levels in the removed paws were measured. All results from the experiment were evaluated by comparing them with the SIC group.

#### 2.4.2 Effects of benidipine, paracetamol, and BPC on the without-incision tissue pain threshold test

For this method, the BN group (n = 8) received benidipine (4 mg/kg) alone by oral gavage, the PC group (n = 8) received paracetamol (500 mg/kg) alone, and the BPCG group (n = 8) 4 mg/kg benidipine plus 500 mg/kg paracetamol. Distilled water was administered as a solvent as in the HC group (n = 8). At the third hour after drug administration, the paw pain thresholds of all rats in all groups were measured using a Basile algesimeter (Cadirci et al., 2010)**.** After the measurements of all groups were completed, the rats were sacrificed with high-dose sodium thiopental (50 mg/kg), and the paw tissues were removed. Oxidant, antioxidant, proinflammatory cytokine, and COX levels were measured in the paws. All results from the experiment were evaluated by comparing them with the SIC group.

### 2.5 Biochemical analysis

Biochemical analyses were performed on the paw tissues of 6 rats randomly taken from each experimental group of 8 rats.

#### 2.5.1 Determination of tissue MDA and tGSH

MDA and total glutathione (tGSH) levels in the tissue specimens were collected using commercial enzyme-linked immunosorbent assay (ELISA) kits manufactured for experimental animals. A 10009055 analysis kit was used for MDA and a 703002 analysis kit for tGSH (Cayman Chemical Company, Ann Arbor, MI, United States).

#### 2.5.2 Measurement of tissue COX activity

COX activity in the rat paw in this series of experiments was determined using a COX activity assay kit (catalogue number: 760151, Cayman Chemical Company). Paw tissue was removed and washed thoroughly with ice-cold Tris buffer, pH 7.4, containing 0.16 mg/mL heparin, to remove any red blood cells and clots and then stored at −80 °C until assayed. A sample of paw tissue from each rat was homogenized in 5 mL of cold buffer (0.1 M Tris-HCl, pH 7.8, containing 1 mM EDTA) per gram of tissue and centrifuged at 10,000 × g for 15 min at 4°C. The resulting supernatant was removed for assay and stored on ice. We then measured the protein concentration in the supernatant by using the Bradford method (Karimi et al., 2022). The COX activity assay kit measures the peroxidase activity of COX. This is assayed colorimetrically by monitoring the appearance of oxidized N, N, N′, N′-tetra methyl-p-phenylenediamine at 590 nm. COX-2 activity was measured using COX-1-specific inhibitor. Results for COX-1 and COX-2 activity are expressed as units per milligram of protein.

#### 2.5.3 Tissue IL-6 analysis

The samples were weighed and trimmed before being rapidly frozen with liquid nitrogen and homogenized by pestle and mortar. They were then maintained at 2°C–8°C after thawing. Phosphate Buffered Saline (pH 7.4) 1/10 (w/v) was added, after which the samples were centrifuged for 20 min at 10,000 × g, at the end of which the supernatants were carefully collected. IL-6 levels (pg/mL) were measured using a commercial ELISA kit (no. SEA079Ra) supplied by Wuhan USCN Business Co. Ltd (Wuhan, Hubei, China).

### 2.6 Statistical analysis

All statistical analyses were performed on IBM SPSS Statistics for Windows version 22.0 software (IBM Corp (2013), Armonk, NY, United States). The results are expressed as mean value ±standard error of the mean (mean ± SEM). The Shapiro-Wilk test was used to determine whether data were normally distributed. One-way analysis of variance was applied to normally distributed data, followed by Tukey’s HSD (Honestly significant difference) test if the homogeneity of variances assumption was met as a *post hoc* test according to the results of Levene’s test, and if not, the Games-Howell test was applied. The Kruskal–Wallis test was applied to non-normally distributed data, and the Mann-Whitney U test was applied as a *post hoc* test. *p*-value of <0.05 were regarded as statistically significant.

## 3 Results

### 3.1 Effects of benidipine, paracetamol, and BPC on postoperative and normal tissue pain thresholds

**FIGURE 1:**
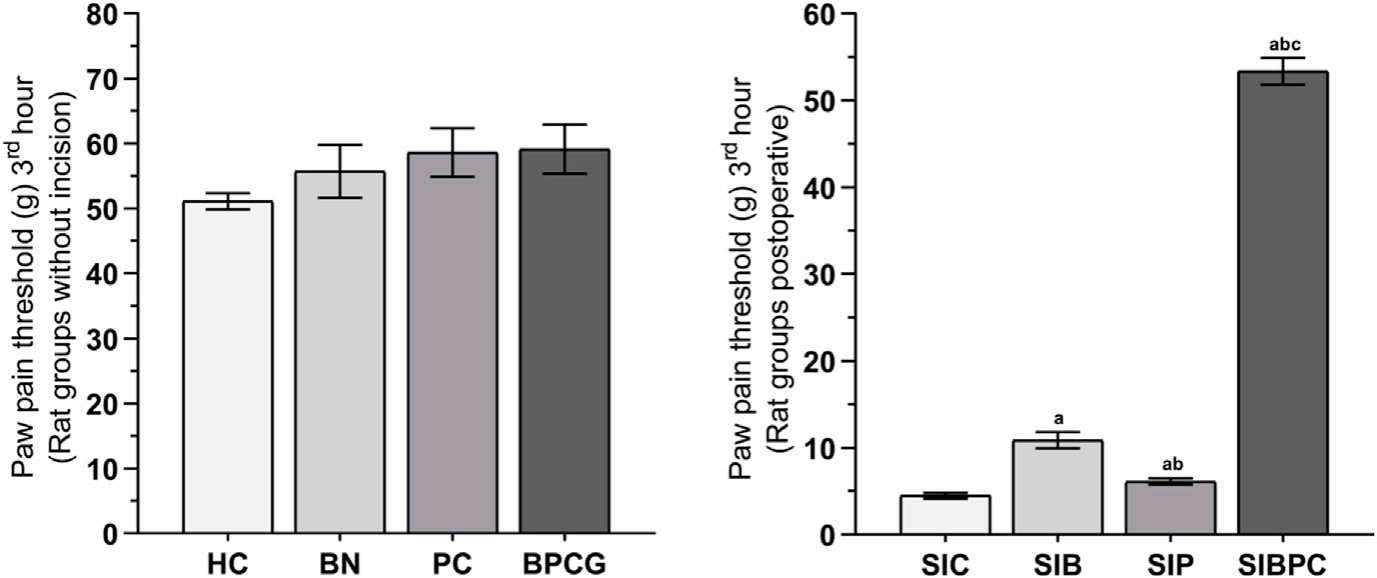
Paw pain threshold at third hours of the without incision and post-operative experimental groups. Footnotes: Bars are mean ± SEM (standard error). ^a^ *p* < 0.05 when all postoperative treatment groups were compared with the SIC control group. ^b^ *p* < 0.05 when the other postoperative drug treatment groups were compared with the SIB alone treatment group. ^c^ *p* < 0.001 when the combined drug treatment group was compared with the SIP alone treatment group (n = 6). Abbreviations: HC: healthy control group; BN: benidipine alone group; PC: paracetamole alone group; BPCG: benidipine and paracetamole combination group; SIC: scalpel incision control group; SIB: scalpel incision + benidipine group; SIP: scalpel incision + paracetamole group; SIBPC: scalpel incision + benidipine and paracetamole combination group.

As shown in Figure 1, benidipine, paracetamol, and BPC produced no significant change in the paw pain threshold of the without-incision animals. Scalpel incision applied to the paw tissue decreased the paw pain thresholds compared to rats in the healthy group (*p* < 0.001). However, while benidipine prevented a decrease in the pain threshold in scalpel-incised paw tissue (*p* = 0.001) better than paracetamol (*p* = 0.020), the best prevention was observed with BPC (*p* < 0.001). As shown in Table 1, the analgesic effects of benidipine, paracetamol, and BPC in the without-incision animals were calculated at 8.3% (*p* = 0.770), 12.8% (*p* = 0.414), and 13.5% (*p* = 0.358), respectively. The analgesic effects of benidipine, paracetamol, and BPC on incised paw tissue were 58.6% (*p* = 0.001), 26.6% (*p* = 0.020), and 91.6% (*p* < 0.001) (Table 2).

**TABLE 1:** Paw pain threshold and analgesic activity values of the rat groups without incision, *post hoc p*-values for group comparisons in the rat groups without incision.

Groups	HC	BN	PC	BPCG
**Paw pain threshold (g) 3**rd **h**	51.13 ± 1.23	55.75 ± 4.05	58.63 ± 3.74	59.13 ± 3.74
**(X±SEM)**				
**Analgesic effect (%)**	-	8.3	12.8	13.5

Groups	HC vs BN	HC vs PC	HC vs BPCG	BN vs PC	BN vs BPCG	PC vs BPCG
*p* values*	0.770	0.414	0.358	0.931	0.894	1.000

**Abbreviations:** HC: healthy control group; BN: benidipine alone group; PC: paracetamole alone group; BPCG: benidipine and paracetamole combination group.

Footnotes: *Tukey HSD, test was performed as the *post hoc* test after one-way ANOVA.

**TABLE 2:** Paw pain threshold and analgesic activity values of the post-operative rat groups, *post hoc p*-values for group comparisons in the post-operative rat groups.

Groups	SIC	SIB	SIP	SIBPC
**Paw pain threshold (g) 3**rd **h**	4.50 ± 0.33	10.88 ± 0.93	6.13 ± 0.35	53.38 ± 1.55
**(X±SEM)**				
**Analgesic effect (%)**	-	58.6	26.6	91.6

Groups	SIC vs SIB	SIC vs SIP	SIC vs SIBPC	SIB vs SIP	SIB vs SIBPC	SIP vs SIBPC
*p* values*	0.001	0.020	<0.001	0.005	<0.001	<0.001

**Abbreviations:** SIC: scalpel incision control group; SIB: scalpel incision + benidipine group; SIP: scalpel incision + paracetamole group; SIBPC: scalpel incision + benidipine and paracetamole combination group.

Footnotes: *Games-Howell test was performed as the *post hoc* test after one-way ANOVA.

### 3.2 Biochemical results

#### 3.2.1 Paw tissue of without-incision groups: MDA and tGSH assay results

**FIGURE 2:**
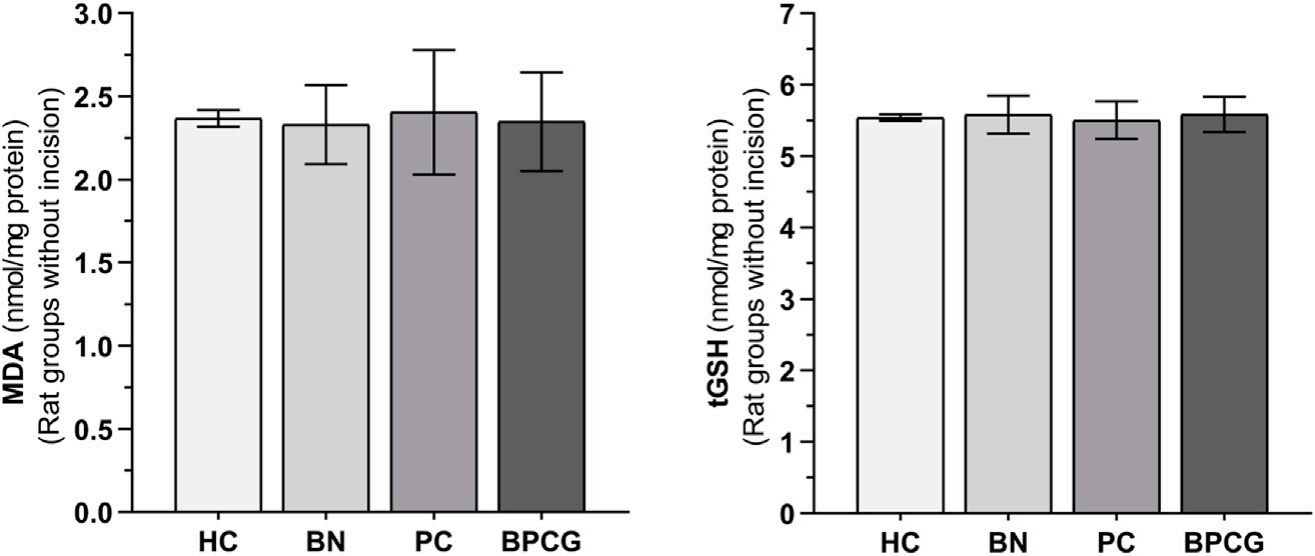
MDA and tGSH levels in foot paw tissues of the without incision experimental groups. Footnotes: Bars are mean ± SEM (standard error). n = 6. Abbreviations: MDA: malondialdehyde; tGSH: total glutathione; HC: healthy control group; BN: benidipine alone group; PC: paracetamole alone group; BPCG: benidipine and paracetamole combination group.

As shown in Figure 2, benidipine, paracetamol, and BPC caused no significant change in MDA (*p* > 0.999 for all) or tGSH (*p* = 0.999 for all) levels in the paw tissue of the without-incision animals (Tables 3 and 4).

**TABLE 3:** Mean and standard error values of the experimental pain test rat groups without incision in terms of foot paw tissue’s biochemical test results.

Groups	MDA	tGSH	COX-1	COX-2	IL-6
**HC**	2.37 ± 0.05	5.54 ± 0.05	7.78 ± 0.03	0.75 ± 0.04	2.20 ± 0.04
**BN**	2.33 ± 0.24	5.58 ± 0.26	7.79 ± 0.52	0.74 ± 0.08	2.11 ± 0.11
**PC**	2.41 ± 0.38	5.51 ± 0.26	7.57 ± 0.49	0.74 ± 0.05	2.18 ± 0.26
**BPCG**	2.35 ± 0.30	5.59 ± 0.25	7.79 ± 0.26	0.74 ± 0.07	2.05 ± 0.12

**Abbreviations:** HC: healthy control group; BN: benidipine alone group; PC: paracetamole alone group; BPCG: benidipine and paracetamole combination group; MDA: malondialdehyde; tGSH: total glutathione; COX-1: cyclooxygenase-1; COX-2: cyclooxygenase-2; IL-6: interleukin-6.

**TABLE 4:** The *p*-values comparison of the experimental pain test rat groups without incision in terms of foot paw tissue’s biochemical test results.

Variable	HC vs BN	HC vs PC	HC vs BPCG	BN vs PC	BN vs BPCG	PC vs BPCG
**MDA**	1.000	1.000	1.000	0.997	1.000	0.999
**tGSH**	0.999	0.999	0.999	0.995	1.000	0.994
**COX-1**	1.000	0.978	1.000	0.976	1.000	0.975
**COX-2**	1.000	1.000	1.000	1.000	1.000	1.000
**IL-6**	0.975	1.000	0.903	0.989	0.993	0.936

**Abbreviations:** HC: healthy control group; BN: benidipine alone group; PC: paracetamole alone group; BPCG: benidipine and paracetamole combination group; MDA: malondialdehyde; tGSH: total glutathione; COX-1: cyclooxygenase-1; COX-2: cyclooxygenase-2; IL-6: interleukin-6.

**Footnotes:** Statistical evaluation was done with one-way ANOVA., afterward, the Tukey HSD, was used as a *post hoc* test.

#### 3.2.2 MDA and tGSH assay results in paw tissue subjected to scalpel incision

**FIGURE 3:**
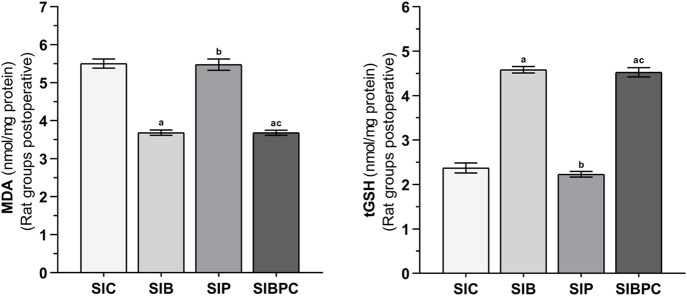
MDA and tGSH levels in foot paw tissues of the postoperative experimental groups. Footnotes: Bars are mean ± SEM (standard error). ^a^ *p* < 0.05 when all treatment groups were compared with the SIC control group. ^b^ *p* < 0.05 when the other drug treatment groups were compared with the SIB alone treatment group. ^c^ *p* < 0.05 when the combination drug treatment group was compared with the SIP alone treatment group. n = 6. Abbreviations: MDA: malondialdehyde; tGSH: total glutathione; SIC: scalpel incision control group; SIB: scalpel incision + benidipine group; SIP: scalpel incision + paracetamole group; SIBPC: scalpel incision + benidipine and paracetamole combination group.

As shown in Figure 3, compared to the HC group, it was observed that tissue MDA levels increased and tGSH levels decreased after claw tissue was cut (*p* < 0.001). Benidipine (*p* < 0.001 and *p* = 0.033, respectively) and BPC (*p* < 0.001 and *p* = 0.042, respectively) significantly prevented an increase in MDA and a decrease in tGSH in paw tissue exposed to scalpel incision, although the effect of paracetamol was non-significant (*p* = 0.999 and *p* = 1.000, respectively) (Tables 5 and 6).

**TABLE 5:** Mean and standard error values of experimental pain test postoperative rat groups in terms of foot paw tissue’s biochemical test results.

Groups	MDA	tGSH	COX-1	COX-2	IL-6
**SIC**	5.50 ± 0.12	2.37 ± 0.11	4.55 ± 0.06	8.57 ± 0.07	5.50 ± 0.04
**SIB**	3.69 ± 0.07	4.58 ± 0.07	6.78 ± 0.04	5.49 ± 0.09	3.13 ± 0.27
**SIP**	5.48 ± 0.15	2.23 ± 0.07	4.12 ± 0.03	8.29 ± 0.33	3.19 ± 0.25
**SIBPC**	3.69 ± 0.07	4.53 ± 0.10	6.62 ± 0.04	5.19 ± 0.04	1.82 ± 0.18

**Abbreviations:** SIC: scalpel incision control group; SIB: scalpel incision + benidipine group; SIP: scalpel incision + paracetamole group; SIBPC: scalpel incision + benidipine and paracetamole combination group; MDA: malondialdehyde; tGSH: total glutathione; COX-1: cyclooxygenase-1; COX-2: cyclooxygenase-2; IL-6: interleukin-6.

**TABLE 6:** The *p*-values comparison of the experimental pain test postoperative rat groups in terms of foot paw tissue’s biochemical test results.

Variable	SIC vs SIB	SIC vs SIP	SIC vs SIBPC	SIB vs SIP	SIB vs SIBPC	SIP vs SIBPC
MDA*	<0.001	0.999	<0.001	<0.001	1.000	<0.001
tGSH**	0.033	1.000	0.042	0.009	1.000	0.012
COX-1***	<0.001	<0.001	<0.001	<0.001	0.092	<0.001
COX-2*	<0.001	0.832	<0.001	0.001	0.068	0.001
IL-6*	0.001	0.001	<0.001	0.998	0.013	0.007

**Abbreviations:** SIC: scalpel incision control group; SIB: scalpel incision + benidipine group; SIP: scalpel incision + paracetamole group; SIBPC: scalpel incision + benidipine and paracetamole combination group; MDA: malondialdehyde; tGSH: total glutathione; COX-1: cyclooxygenase-1; COX-2: cyclooxygenase-2; IL-6: interleukin-6.

**Footnotes:** *Games-Howell test was performed as the *post hoc* test after one-way ANOVA, *Mann-Whitney U test was performed as the *post hoc* test after Kruskal–Wallis test, ***Tukey HSD, test was performed as the *post hoc* test after one-way ANOVA.

#### 3.2.3 Paw tissue of groups without incision: COX-1, COX-2, and IL-6 assay results

**FIGURE 4:**
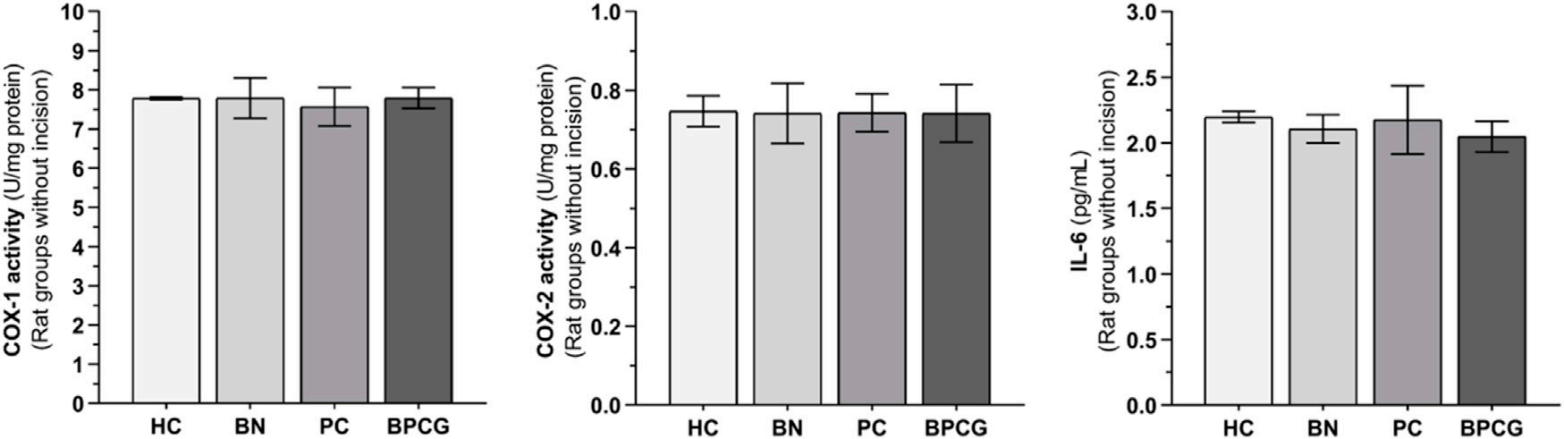
COX-1, COX-2, and IL-6 levels in foot paw tissues of the without incision experimental groups Footnotes: Bars are mean ± SEM (standard error). n = 6. Abbreviations: COX-1: cyclooxygenase-1; COX-2: cyclooxygenase-2; IL-6: interleukin-6; HC: healthy control group; BN: benidipine alone group; PC: paracetamole alone group; BPCG: benidipine and paracetamole combination group.

Benidipine, paracetamol, and BPC caused no significant changes in the without-incision animals’ paw tissue COX-1 (respectively *p* > 0.999; *p* = 0.978; *p* > 0.999), COX-2 (*p* > 0.999 for all), or IL-6 (respectively *p* = 0.975; *p* > 0.999; *p* = 0.903) levels (Figure 4). The differences between these groups were also nonsignificant (Tables 3 and 4).

#### 3.2.4 COX-1, COX-2, and IL-6 assay results in paw tissue exposed to scalpel incision

**FIGURE 5:**
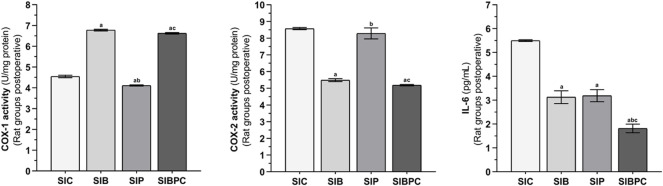
COX-1, COX-2, and IL-6 levels in foot paw tissues of the postoperative experimental groups. Footnotes: Bars are mean ± SEM (standard error). ^a^ *p* < 0.001 when all treatment groups were compared with the SIC control group. ^b^ *p* < 0.05 when the other drug treatment groups were compared with the SIB alone treatment group. ^c^ *p* < 0.05 when the combination drug treatment group was compared with the SIP alone treatment group. n = 6. Abbreviations: COX-1: cyclooxygenase-1; COX-2: cyclooxygenase-2; IL-6: interleukin-6; SIC: scalpel incision control group; SIB: scalpel incision + benidipine group; SIP: scalpel incision + paracetamole group; SIBPC: scalpel incision + benidipine and paracetamole combination group.

As indicated in Figure 5, an increase in tissue COX-2 activity and IL-6 levels and a decrease in COX-1 activity were observed after claw tissue was cut compared to the HC group (*p* < 0.001). Benidipine (*p* < 0.001 for both) and BPC (*p* < 0.001 for both) significantly prevented a fall in COX-1 and a rise in COX-2 in paw tissue exposed to scalpel incision. However, while paracetamol exhibited a significant inhibitory effect on COX-1 (*p* < 0.001), its inhibitory effect on COX-2 was nonsignificant (*p* = 0.832). In addition, benidipine (*p* = 0.001) and paracetamol (*p* = 0.001) exhibited almost equal prevention of IL-6 elevation in paw tissue with scalpel incision. However, the best suppression of IL-6 elevation in paw tissue with scalpel incision was exhibited by BPC (*p* < 0.001) (Tables 5 and 6).

## 4 Discussion

This study investigated the effects of benidipine, paracetamol, and BPC on normal tissue pain thresholds experimentally induced in rats. The experimental results showed that the paw pain threshold in the group exposed to scalpel incision decreased significantly compared to the without-incision (normal tissue) group. In support of our results, Cetin et al. also reported that scalpel incision significantly lowered the pain threshold (Cetin et al., 2016). Information in the literature suggests that reactive oxygen species (ROS) that increase during the postoperative process cause oxidative stress and lead to pain by impairing antioxidant balance (Karkkainen et al., 2018). In the present study, MDA was measured in the evaluation of pain because it is a toxic product of lipid peroxidation and an important marker of oxidative stress (Abbaszadeh et al., 2018). Previous studies have also reported that an increase in MDA levels leads to hypersensitivity to pain (da Silva et al., 2021). Ince et al. reported a significant increase in MDA levels in the paws of rats exposed to scalpel incision (Ince et al., 2015). The higher MDA levels in the scalpel-incision group compared to those in the HC group in the present study indicates that our experimental results are consistent with the previous literature.

Measurement of changes in antioxidant levels is one frequently employed method for elucidating the pathology of pain developing in association with ROS in the postoperative period (Ince et al., 2015; Onk et al., 2018). Levels of tGSH, a principle endogenous antioxidant, were therefore measured in the present study. GSH, a low molecular weight tripeptide, protects cells against oxidative damage by reacting with ROS and peroxides (Guo et al., 2018). As shown by our findings, tGSH levels decreased in paw tissue after scalpel incision compared to the HC group. Consistent with our experimental results, Cetin et al. showed that scalpel incision caused a decrease in tGSH in rat paw tissue (Cetin et al., 2016).

ROS, the production of which increases in the postoperative period, also increase the production of prostaglandins in the same tissue (Rahmanian-Devin et al., 2021). Prostaglandins stimulate C-fibre pain receptors and cause pain by lowering the stimulation thresholds of polymodal receptors (Zeilhofer, 2007; Jang et al., 2020). The levels of the enzymes COX-1 and COX-2 involved in the synthesis of prostaglandins were therefore measured in the present study in order to evaluate pain. COX-1 is a structural enzyme responsible for a protective effect, while COX-2 is an inducible enzyme responsible for inflammatory events (Suleyman and Ozcicek, 2019). Decreased COX-1 has been linked to increased inflammation and pain sensitivity (Zeilhofer, 2007), while increased COX-2 has been linked to postoperative inflammation (Schug, 2006; Gao et al., 2020). Additionally, an increase in COX-2 levels has been reported to be associated with increased nociceptor sensitivity and hyperalgesia (Jang et al., 2020Ince et al., 2015). In agreement with the previous literature, COX-1 activity in rat paw tissues with scalpel incision was lower than that in the control group in the present study, while COX-2 activity was higher.

Overproduction of proinflammatory cytokines in tissue during the postoperative process is known to play an important role in the persistence of pain (Gao et al., 2020). The proinflammatory cytokine IL-6 contributes to inflammatory response manifestations by interacting with neurons along the pain pathway (Manjavachi et al., 2010; Moy et al., 2017). IL-6 released following tissue injury in the postoperative period has been reported to increase the sensitivity of nociceptors and to potentialize pain perception (Chen et al., 2013; Kummer et al., 2021). Additionally, increasing IL-6 has been shown to enhance nociceptor sensitivity and initiate pro-algesic effects (Opree and Kress, 2000). Our tissue analysis results exhibited a significant increase in IL-6 levels in the scalp incision group compared to the without-incision group.

BPC, whose analgesic effect was investigated in this study, raised the pain threshold in paw tissue with scalpel incision more than benidipine or paracetamol applied alone. However, the effects on the pain threshold with benidipine, paracetamol, and BPC applied to the without-incision rats were nonsignificant. Despite the presence of information in the literature to the effect that paracetamol significantly raises the pain threshold in rats with scalpel incisions (Ince et al., 2015), we encountered no studies investigating the relationship between benidipine and postoperative pain. However, a case report stated that benidipine eliminated nivolumab-induced angina pectoris pain (Kumamoto et al., 2022). Our findings indicate that benidipine and paracetamol in combination more significantly suppress hypersensitivity in nociceptors by exhibiting a additive effect.

The effects of benidipine, paracetamol, and BPC on MDA and tGSH in the without-incision rats were nonsignificant. Benidipine significantly prevented an increase in MDA and decrease in tGSH in the postoperative period in rats with scalp incision, while paracetamol did not prevent these. Information suggesting that pain is associated with an increase in intracellular calcium and MDA (da Silva et al., 2021) partly explains the analgesic effect mechanism of benidipine. Benidipine is known to exhibit an antioxidant effect (Cakir et al., 2021). However, paracetamol exhibited no significant effect on oxidant and antioxidant parameters in rats with scalp incisions in paw tissues (Ince et al., 2015). Our findings suggest that paracetamol may possess a different analgesic effect mechanism than the inhibition of oxidative stress.

The drugs tested in this study had no significant effect on COX-1 or COX-2 in the without-incision rats. Benidipine alone and BPC significantly prevented a decrease in COX-1 and an increase in COX-2 in tissue exposed to scalpel incision. COX-1 levels decreased in paw tissue with scalpel incision in the paracetamol group, but no significant effect was observed on COX-2 levels. Our scan of the literature yielded no information concerning the effects of benidipine on COX-1 and COX-2 levels in postoperative tissue. However, previous studies have reported that an increase in COX-2 in the postoperative period is associated with an increase in intracellular calcium (Moy et al., 2017; Guo et al., 2018). In addition, benidipine was reported to significantly inhibit the decrease in COX-1 activity and the increase in COX-2 activity in the rat liver and to protect the liver against ischaemia-reperfusion injury (Cimen et al., 2019). The fact that benidipine is a calcium channel antagonist suggests that it inhibits an increase in COX-2 activity by decreasing intracellular calcium concentrations. Our determination of the analgesic effect of paracetamol in incised tissue indicates that it provides analgesia through a different mechanism than COX-2 inhibition.

Benidipine and paracetamol significantly inhibited an increase in IL-6 production in the paw tissue of rats exposed to scalpel incision. However, BPC reduced the increase in IL-6 more significantly than benidipine or paracetamol. At the same time, benidipine, paracetamol, and BPC had no significant effects on IL-6 levels in the without-incision rats. Our scan of the literature elicited no information about the relationship between benidipine or paracetamol and IL-6 in postoperative pain. However, Nakamura et al. reported that treatment with benidipine exhibited an anti-inflammatory effect by stabilizing the production of the proinflammatory cytokine IL-6 in acute kidney failure (Nakamura et al., 2000). Additionally, paracetamol has been reported to significantly reduce serum IL-6 levels in the postoperative period (Garrone et al., 2021) and in febrile patients (Honarmand et al., 2012). Considering that IL-6 increase induces pain generation (Chen et al., 2013; Kummer et al., 2021), the additive effects of the combination of benidipine and paracetamol on the normalisation of tissue IL-6 levels seem to increase analgesic activity.

Although paracetamol is a well-tolerated drug that causes few side effects in the gastrointestinal tract, its effectiveness in relieving pain has been demonstrated in a limited number of diseases and its benefits have generally been modest (Jóźwiak-Bebenista and Nowak, 2014; Abdel Shaheed et al., 2021). Additionally, paracetamol-induced liver toxicity is a worldwide concern (Jóźwiak-Bebenista and Nowak, 2014). As for benidipine, toxicity studies and clinical reports show that it is a safe drug over a wide range of doses. However, no study on the possible toxicity of the combination of paracetamol and benidipine could be found in the literature review and this issue needs to be investigated (Yang et al., 2019).

## 5 Limitations

The measurement of both proinflammatory and anti-inflammatory cytokine levels is important for a more detailed clarification of the analgesic effect mechanism. In addition to determining paw pain thresholds, it is also important to include different evoked pain behavior tests and ongoing pain behavior tests. The fact that the sciatic nerve, dorsal root ganglion and spinal cord tissues related to the pain pathway were not analysed molecularly is among the limitations of this study.

## 6 Conclusion

Benidipine, paracetamol, and BPC produced no significant change in the without-incision animal group paw pain threshold or in oxidant, antioxidant, COX-2, and IL-6 levels. However, these drugs significantly prevented a fall in the pain threshold in paws subjected to scalpel incision. BPC best prevented a decrease in paw pain threshold, followed by benidipine, and finally paracetamol. In addition, increases in MDA, COX-2, and IL-6 and a decrease in tGSH in tissue with scalpel incision were significantly suppressed by benidipine and BPC, although paracetamol was only capable of inhibiting an increase in the production of IL-6. The analgesic effect of benidipine may derive from its more powerful inhibitory effect on MDA, COX-2, and IL-6 and antioxidant activity compared to paracetamol. BPC provided more powerful analgesia than benidipine or paracetamol alone. This may be due to the additive inhibitory effects of benidipine and paracetamol on IL-6. Furthermore, our study results support that the combination we used produces analgesic activity through both common and different analgesia mechanisms. Our experimental results suggest that BPC may be useful in the treatment of severe pain that is not sufficiently suppressed by paracetamol.

## Data Availability

The original contributions presented in the study are included in the article/Supplementary Material, further inquiries can be directed to the corresponding author.
