# Supplementary figures and images for: Effects of benidipine, paracetamol, and their combination on postoperative and normal tissue pain thresholds
Source: Front Pharmacol. 2024 Jan 5;14:1326128. doi: 10.3389/fphar.2023.1326128 (PMC10796563; doi:10.3389/fphar.2023.1326128)

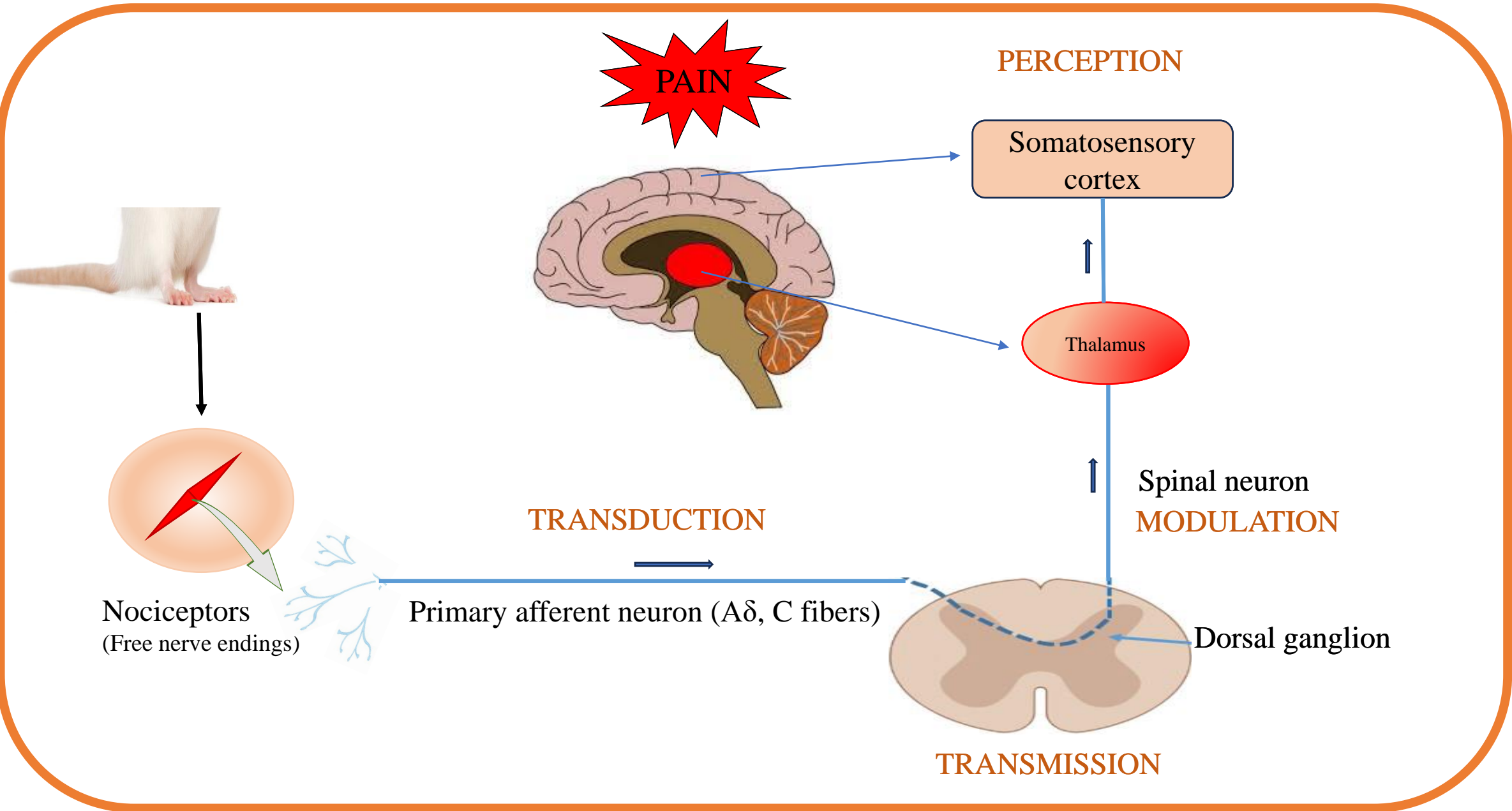

Supplement: Supplementary file 1 [file DataSheet1.PDF]
